# Apple Derived Exosomes Improve Collagen Type I Production and Decrease MMPs during Aging of the Skin through Downregulation of the NF-κB Pathway as Mode of Action

**DOI:** 10.3390/cells11243950

**Published:** 2022-12-07

**Authors:** Martina Trentini, Ilaria Zanolla, Federica Zanotti, Elena Tiengo, Danilo Licastro, Simeone Dal Monego, Luca Lovatti, Barbara Zavan

**Affiliations:** 1 Department Translational Medicine, University of Ferrara, 44121 Ferrara, Italy; 2 AREA Science Park, Padriciano 99, 34149 Trieste, Italy

**Keywords:** apple, inflammageing, exosomes, extracellular matrix, wound healing

## Abstract

Skin ageing is strictly related to chronic inflammation of the derma and the decay of structural proteins of the extracellular matrix. Indeed, it has become common practice to refer to this phenomenon as inflammageing. Biotech innovation is always in search of new active principles that induce a youthful appearance. In this paper, apple-derived nanovesicles (ADNVs) were investigated as novel anti-inflammatory compounds, which are able to alter the extracellular matrix production of dermal fibroblasts. Total RNA sequencing analysis revealed that ADNVs negatively influence the activity of Toll-like Receptor 4 (TLR4), and, thus, downregulate the NF-κB pro-inflammatory pathway. ADNVs also reduce extracellular matrix degradation by increasing collagen synthesis (COL3A1, COL1A2, COL8A1 and COL6A1) and downregulating metalloproteinase production (MMP1, MMP8 and MMP9). Topical applications for skin regeneration were evaluated by the association of ADNVs with hyaluronic-acid-based hydrogel and patches.

## 1. Introduction

In dermatology, a youthful appearance is attained through treatments promoting skin rejuvenation. However, cutaneous ageing will occur by environmental and intrinsic physiological factors [1]. To maintain its integrity and elasticity, this organ is especially good at regenerating itself at each layer: epidermis, dermis and hypodermis. Dermis decaying composition, structure and organization is the cause of wrinkles, skin sagging and other signs of skin ageing. In children and adults, it is rich in connective tissue, which is composed of fibroblasts and extracellular matrix (ECM) [2]. The ECM is gel-like, and it is comprised largely of proteins, such as collagen, elastin and laminin, and associated with proteoglycans and glycosaminoglycans. Its composition is ideal to support cellular migration, proliferation and adhesion [3,4].

Upon ageing, the tissue draws mitochondrial dysfunction, deregulated nutrient sensing, epigenetic alterations, telomere attrition, genomic instability and altered intercellular communication [5,6]. These events lead to senescent cells and stem cell exhaustion. Fibroblasts produce less ECM while increasing the transcription of metalloproteinase (MMP) and decreasing tissue inhibitors of metalloproteinase synthesis (TIMP) [7]. The low turnover of the ECM causes a loss of dermal elasticity.

Another reason for elasticity loss is damage to the ECM. Oxidative stress is a pivotal biochemical event inducing degradation and disorganization in the ECM [1]. UV radiations are common initiators of reactive oxygen species (ROS) generation in skin, which cause cell and ECM damage, targeting the collagen network and elastin (elastosis) in a process called photoaging. In the skin, keratinocytes and fibroblasts are the leading producers of mitochondrial ROS; therefore, ageing-induced mitochondrial dysfunction also endangers fibroblasts and fibroblast-produced ECM.

During cutaneous ageing, senescent and radical-damaged cells trigger inflammation and erythema [8,9,10,11,12,13]. Inflamed cells produce growth factors, pro-inflammatory cytokines and chemokines, whose role is to chemoattract and activate cells of the innate immune system. On site, phagocytes and natural killers eliminate damaged cells and molecules. However, uncontrolled inflammation can produce counter-effective results. Tissue damage can be due to an overly stimulated innate immune system and an exaggerated production of chemokines and cytokines. This often occurs in older skin, as tissue-specific immune surveillance decreases with people’s age [7].

The field of regenerative medicine is therefore in constant search of new antioxidant and immunomodulatory therapeutics for skin regeneration [14,15]. Rejuvenation therapies that have been investigated include direct applications of mesenchymal stem cells, raising quite a few safety issues [16,17]. A novel “cell-free” therapy with similar results concerns the use of MSC-derived extracellular vesicles [18,19,20,21,22,23,24]. Vesicles of nanometric proportions (NVs) are a physiological part of complex intercellular signaling [25] A double lipid layer surrounds a bioactive cargo loaded in the cell of origin and delivered in the recipient cell as a message. The outer layer mirrors the cell-type-specific membrane, while cargo molecules are cytosolic components, mainly nucleic acids and proteins, that trigger the recipient cells. Extracellular vesicles have been categorized based on their size [26]. The most commonly studied are exosomes, vesicles of 50–150 nm in diameter originating from the cytosolic multi vesicular body (MVB) in mammalian cells [27]. Exosomes have been investigated as diagnostic and therapeutic tools and as drug-delivery systems with cell-specific target capabilities [28,29,30]. In a study by Xu et al. (2020), MSCs-NVs were subcutaneously injected in photoaged mice, resulting in lower ROS generation and increased cellular proliferation of fibroblasts [31]. In recent years, investigators have discovered that NVs also play a role in vegetable communication.

Plant-derived NVs have been successfully isolated and characterized from several edible fruits and vegetables, including lemon [32], strawberry [33], grape [34], ginger [35] and broccoli [36]. Interestingly, several investigations showed how plant-derived NVs can interact and influence mammalian cells, indicating the possibility of cross-kingdom communications [37,38,39]. Several NVs have exhibited antioxidant and anti-inflammatory properties. An in vivo study by Deng et al. (2017) observed broccoli-derived NVs’ anti-inflammatory action by preventing dendritic cell activation and protecting mice against colitis [36]. Citrus-derived NVs observed in vitro relieved MSCs from oxidative stress [32], while in vivo they inhibited cancer cell proliferation and cell viability through the activation of TRAIL-mediated apoptosis and reduction of pro-angiogenic cytokines IL-6 and IL-8 [40].

Apples, one of the most consumed fruits globally, are rich in polyphenols, whose exact composition is variety-dependent [41,42]. Polyphenols are anti-cancer, anti-inflammatory and antioxidant molecules [43]. Thanks to their high content in polyphenols, and to the famous saying, apples are already perceived as a healthy fruit by the general population. Apple-derived nanovesicles (ADNVs) have been isolated and characterized. Their range is approximately around 130 nm in diameter. They have exhibited anti-inflammatory activity in canonically activated THP-1-derived macrophages by the regulation of miR-146a [44]. On the strength of these findings, the research community is investigating plant-derived NV potential and exploiting them for several applications [45].

This study aimed to investigate ADNVs’ effect on human cells involved in skin ageing and reparation. ADNVs were associated with well-known cosmetic delivery systems to investigate possible applicability issues, all the while looking for the molecular markers predictive of ADNVs’ effect on human cell lines.

## 2. Materials and Methods

### 2.1. EV Isolation and Quantification

Apple-derived nanovesicles (ADNVs) were isolated from DOP Golden delicious variety apples (*Malus domestica* sp.) cultivated in Val Di Non, Trentino (Italy). Briefly, three apples of approximately 250 g each were washed thoroughly and smashed into a pulp. The pulp was subjected to a series of centrifugations at increasing speed (650× *g*, 3000× *g* and 10,000× *g*). After each spin, the pellet was discharged and the supernatant retained. The supernatant fraction was then filtered with 0.22 μm syringe filters (GVS S.p.A, Rome, Italy) and centrifuged at 15,000× *g* with Ultracentrifuge Optima L-70 (Beckman Coulter Inc., Pasadena, CA, USA) type 70 Ti rotor to remove smaller particles and debris. The supernatant was further centrifuged at 110,000× *g*. The pellet, resulting from this last centrifugation, was resuspended in 1 mL of PBS (Thermo Fisher Scientific, Waltham, MA, USA) and was used as the ADNV fraction in all following experiments. The ADNV fraction was conserved at −80 °C until use.

### 2.2. ADNV Quantification and Size Characterization

Following isolation, ADNVs in PBS were quantified through tunable resistive pulse sensing (qNANO Gold, Izon Science Ltd., Cambridge, MA, USA). The analysis also provided data on size distribution of particles in the ADNV fraction. The nanopore (NP150, Izon Science Ltd., Cambridge, MA, USA) was stretched 49 mm wide using a digital caliber. The preparation of the instrument was performed with reagents provided by the manufacturer following the manufacturer’s instructions. Each sample was analyzed at two pressure points, 10 atm and 20 atm. During each measurement, the particle rate was maintained above 200 particles/min, and the total particle count surpassed 500 particles. Calibration particle (CPC200, Izon Science Ltd., Cambridge, MA, USA) measurements were taken at both 10 atm and 20 atm and were used for calibrating each sample measurement during data analysis. The protein content was also established through Bradford assay with Pierce™ BCA Protein Assay Kit (Thermo Fisher Scientific, Waltham, MA, USA) following manufacturer’s instructions. Absorbance was measured at 570 nm using multilabel plate reader Victor 3 (Perkin Elmer, Milano, Italy).

### 2.3. SEM Imaging of ADNVs

To assess ADNV morphology, electronic imaging techniques were put into practice. After isolation, ADNVs were resuspended in 1 mL of 2% glutaraldehyde in phosphate buffer for fixation. The fixed fraction particles were allowed to gravity settle for 1 h on a clean cover slide at room temperature (25 °C). For observation under scanning electron microscopy (SEM), the sample was dehydrated by washes with increasing ethanol concentrations (50%, 70%, 80%, 90%, 100%). The coverslip was mounted on proper support and sputter-coated with gold following standard procedures. Imaging was performed under high vacuum condition using secondary electron detector with SEM Zeiss EVO 40 (Zeiss, Oberkochen, Germany).

### 2.4. MeHA and HY Release Assays

A gel-like solution of 2% hyaluronic acid (HA 2%) was used as the base for the addition of ADNVs. To create the final formulation, 20 μL of the ANDV fraction was mixed with 1 mL of Hyaluronic acid 2% gel for a final concentration of 500 μg/mL. 3D-printed methacrylate hyaluronic acid patches (MeHA) were re-hydrated with 500 μL of ADNV solution (500 μg/mL) [46]. ADNVs’ release from the hyaluronic acid formulation and MeHA was monitored by measuring the protein concentration in the release reservoir. Briefly, 0.1 mL of hyaluronic acid w/ ADNVs was placed in a 48 multi-well and covered with 0.1 mL of PBS, which constituted the reservoir. Replicates of the experiment were kept at room temperature (25 °C) or at physiological temperature (37 °C). Every two hours over a period of 12 h, the reservoir was removed and replaced by 0.1 mL of fresh PBS. Each fraction was kept at 4 °C until all were collected. Similarly, a 0.5 × 0.5 mm square MeHA w/ ADNVs was placed in a 48 multi-well and covered with 0.1 mL of fresh PBS as reservoir. The analysis was carried on as described for HY w/ ADNVs. Protein quantification was performed on each reservoir fraction with Bradford assay, as described in the Section 3.1. All experiments were performed in triplicates.

### 2.5. Cell Culture and Treatment

Human primary dermal fibroblasts were purchased from Resnova (Rome, Italy, from old donor). Fibroblasts were maintained as monolayer cultures in 75 mL flasks (Corning, VWR) in an incubator at 37 °C, 5% CO_2_ and 80% humidity. Cells were passaged every 72 h, dissociated with TrypLE^TM^ (Thermo Fisher Scientific, Waltham, MA, USA) and spun at 265× *g*. Medium was aspirated, and cells were resuspended in pre-warmed DMEM (Euroclone S.p.a, Milan, Italy) containing 10% FBS (Euroclone S.p.a, Milan, Italy). Once 70% confluency was reached, inflammation was induced in fibroblasts by the addition of TNFα (10 ng/mL) (Thermo Fisher Scientific, Waltham, MA, USA) to complete DMEM, for six hours. Cells cultured both w/ or w/o TNFα were treated with ADNVs for 24 h and also added to complete DMEM (protein concentration 200 μg/mL). For RT-qPCR experiment were used two ADNV concentrations: 1× (200 μg/mL) and 0.5× (100 μg/mL) for 48 h.

### 2.6. Migration Assay-Scratch Test

Cells were cultured to 100% confluency on a 6-well plate either w/ or w/o TNFα, as mentioned above. The scratch test was performed as described by Chun Chi Liang and Ann Y. Park et al. (2007) [47]. Briefly, with a 200 μm tip, a straight scratch was performed in the middle of the well, removing cells in that area. Wells were then washed three times with PBS to remove debris, and new medium was added to the cells for treated and control conditions (w/ or w/o ADNVs, respectively). The scratches were marked to ensure that images were taken in the same position and, thus, cell migration progress could be monitored evenly. Images were acquired with EVOS cell imaging system inverted microscope (Thermo Fisher Scientific, Waltham, MA, USA) every 2 h for 12 h. For each condition, the experiment was performed in triplicate, each comprised of twelve replicates.

### 2.7. Cell Viability-MTT Assay

MTT assay was performed on NCTC L929 cells after ADNV incubation. Briefly, all samples were incubated with 1 mL of 0.5 mg/mL MTT [3-(4,5-dimethythiazol-2-yl)-2,5-diphenyl tetrazolium bromide] solution in PBS for 3 h at 37 °C. The MTT solution was then removed, and the formazan cell content was extracted with 0.5 mL of 10% DMSO. OD values were recorded at 570 nm for each sample in duplicate, using a multilabel plate reader (Victor 3, Perkin Elmer, Milano, Italy) [48]. This experiment was performed three times independently.

### 2.8. Evaluation of Reactive Oxygen Species Production

MitoSOX^TM^ fluorescent probe (Thermo Fisher Scientific, Waltham, MA, USA) superoxide indicator was used to assess the level of oxidative stress in fibroblasts w/ and w/o TNFα, w/ or w/o ADNVs. After culture and treatment, cells were washed with PBS. The assay was carried out according to manufacturer’s instructions. After incubation with the fluorescent dye, cells were resuspended in 100 μL of PBS. Red fluorescence was read with Tali^TM^ image-based cytometer (Invitrogen, Thermo Fisher Scientific, Waltham, MA, USA), with 13 fields capture. Fluorescence unit (RFU) threshold was applied at 1000 RFU for all samples. All experiments were carried out in triplicate.

### 2.9. RNA Extraction and RT-qPCR

Fibroblasts cultured w/ or w/o TNFα and w/ or w/o ADNVs underwent lysis. Total RNA was extracted using Total RNA Purification Plus Kit (Norgen Biotek Corp., Thorold, ON, Canada) following manufacturer’s instructions for cells growing in monolayer. The extracted RNA quality and concentration was verified with NanoDrop One (Thermo Fisher Scientific, Waltham, MA, USA). RNA was then stored at −80 °C until use. First strand cDNA was synthetized from total RNA. For each sample, 1200 ng of RNA were reverse-transcribed with SensiFAST cDNA Synthesis Kit (Meridian Bioscience, Boston, MA, USA) in a final volume of 20 μL. Forward and reverse primers for genes of interest (GOI) and housekeeping genes (HKG) (Table 1) were added to sample’s cDNA and SensiFAST SYBR No-ROX master mix (Meridian Bioscience, USA) according to manufacturer’s instructions. Real-time quantitative PCR was performed with GDS Rotor-Gene^®^ Q Thermocycler (Qiagen, London, UK). Thermal cycling and fluorescence detection were carried out as follows: PCR activation step at 95 °C for two minutes, followed by 40 repeating cycles of denaturation (95 °C for 5 s), annealing (60 °C for 10 s) and extension (70 °C for 20 s). A melting step was added at the end, with temperatures gradually increasing from 72 °C to 95 °C over five minutes. Three biological replicates were used for each sample, and three technical replicates were measured.

### 2.10. Total RNA Sequencing

Total RNA was quantitatively and qualitatively evaluated using NanoDrop 2000 (Thermo Fisher Scientific, Waltham, MA, USA) and Agilent Bioanalyzer 2100 (Agilent, Santa Clara, CA, USA), respectively. Libraries were generated with 1 μg of total RNA by TruSeq Sample Preparation RNA Kit (Illumina Inc., San Diego, CA, USA) according to the manufacturer’s protocol. The quality and quantity were analyzed with Bioanalyzer 2100 (Agilent Technologies, Berlin, Germany) on DNA 1000 Chip. The libraries were quantified using Qubit dsDNA BR Assay Kit (Thermo Fisher Scientific, Waltham, MA, USA) on Qubit 2.0 Fluorometer (Thermo Fisher Scientific, Waltham, MA, USA). Sequencing was performed on Novaseq 6000 sequencer (Illumina Inc., San Diego, CA, USA) according to the manufacturer’s protocol. Illumina BCLFASTQ v2.20 software was used for de-multiplexing and the production of FASTQ files. Raw files were subsequently quality checked with FASTQC software (http://www.bioinformatics.bbsrc.ac.uk/projects/fastqc; accessed on 15 October 2022), and sequences with low quality score or including adaptor dimers were discarded from the analysis. The resulting set of selected reads were aligned onto the complete human genome using Splices Transcripts Alignment to a Reference algorithm STAR version 2.7.3 using hg38 Genome Assembly and Genecode.v35 as gene definition. The resulting mapped reads were used as input for feature count functions of Rsubread packages and used as gene counts for differential expression analysis using Deseq2 package [49]. Reads for fibroblasts grown w/o TNFα were confronted with fibroblasts grown w/o TNFα and w/o ADNVs, while fibroblasts treated w/ TNFα were confronted with fibroblasts treated w/ both TNFα and ADNVs. Differentially expressed genes (DEGs) were selected for log_2_(FC) < −1 or >1 and Adjp-value < 0.05.

### 2.11. Bioinformatic and Statistical Analysis

All results were represented as mean, with an indication of the standard error (SE) obtained from at least three independent replicas of the experiment. Significant difference between groups was determined by ANOVA analysis of variance and multiple comparisons post hoc Bonferroni test with Prism 8.03 software (GraphPad Software Inc, Boston, MA, USA). Statistical significance is labelled as follows: * *p* < 0.05, ** *p* < 0.01, *** *p* < 0.001 and **** *p* < 0.0001. Images from the migration test were analyzed with ImageJ software, the distance between cells was measured with the Measure tool, and the difference through time was calculated with Microsoft Excel. All datasets from RNA sequencing were analyzed with Qiagen Ingenuity Pathway Analysis (IPA) software core analysis. Through IPA, all DEGs were categorized in canonical pathways. IPA also predicted possible master regulators and downstream disease and functions. “Upstream regulators” were ranked based on their significance (*p*-value) and predicted state of activation/inhibition (z-Score). IPA assorts plausible downstream effects derived from (i) the activation/inhibition of upstream regulators and (ii) the DRGs expression rate in “Diseases and Functions”. Upstream and downstream predictions and DEGs were combined in functional networks. At each prediction, IPA attributes a z-Score, which was cut off at z-Score < −2 or >+2. Heatmaps were used to demonstrate the expression patterns of these DEGs. Data were cross-checked with KEGG database for interpretation and visual aid.

## 3. Results

### 3.1. Quantification and Characterization of ADNVs

**Figure 1:**
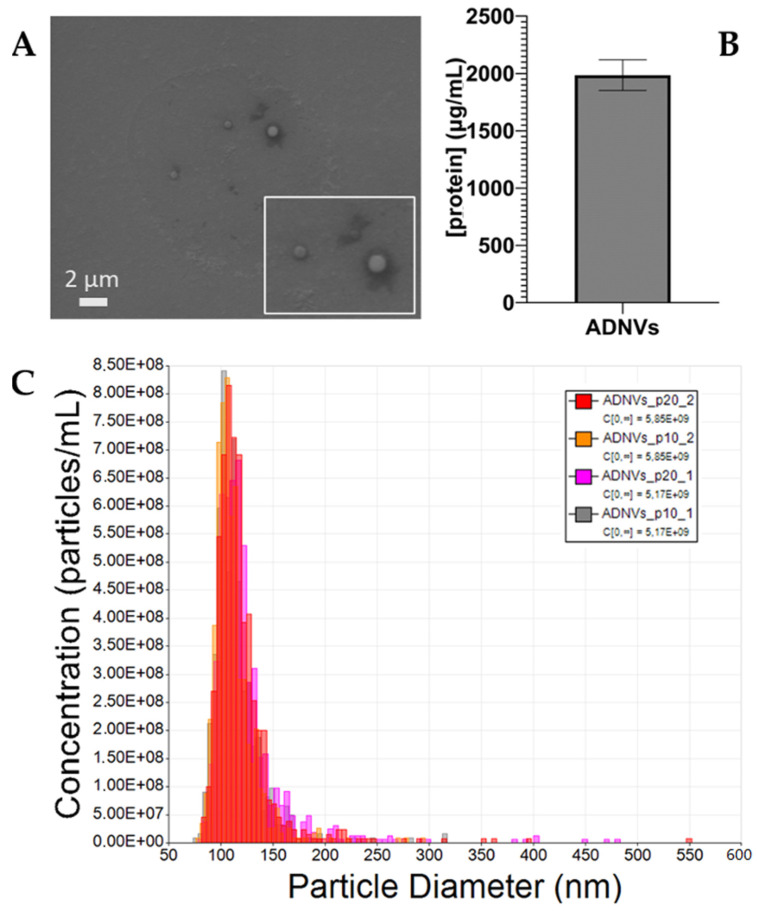
(**A**) SEM Imaging of ADNVs (MAG, 5.0 KX; WD, 15 mm; EHT, 15 kV; High Vacuum); (**B**) Protein quantification of the ADNV fraction after isolation (μg/mL); and (**C**) TRPS analysis output.

In this study, ADNVs isolated from the Golden delicious apple variety were identified by means of electron microscopy imaging, quantified by protein content and physically characterized through TRPS. ADNV morphology was examined through scanning electron microscopy (Figure 1A), where it was clearly reported that they are visible as round-shaped objects, ranging from 80 nm to 500 nm, in accordance with TRPS dimension analysis. The quantification of ADNVs, in order to associate them with TRPS, was based on the protein content in the sample. On average, the protein concentration amounted to 2000 ng/mL (Figure 1B), meaning that 200 μg/mL of proteins corresponded to 4456.7 particles/mL. Lastly, TRPS quantification and size distribution analysis outcomes are shown in Figure 1C, while details on each run are shared in Table 2. The overlapping histograms, each referring to the same replicate measured at two different pressures (20 atm and 10 atm), demonstrate the accuracy of the single measurement. The particle sizes range from 80 nm to 300 nm, with a few outliers up to 550 nm. The most densely populated size range is between 100 nm and 120 nm, with an average diameter of 115–120 nm. The raw particle concentration in the ADNV fraction stands around 5.5 × 10^9^ particles/mL.

### 3.2. EVs’ Release from HA Formulation and MeHA

**Figure 2:**
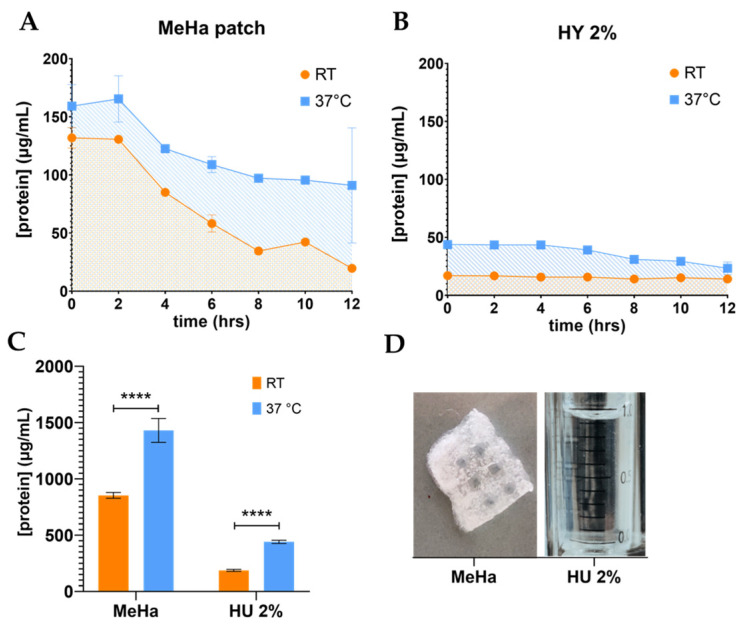
(**A**,**B**) Release curve of ADNVs from hyaluronic-acid-based materials, MeHA patch and HY 2% gel, at room temperature (RT = 25 °C) and physiological temperature (37 °C). ADNV release is expressed in form of protein concentration (μg/mL) in time (hours). (**C**) Values corresponding to the area underneath each curve in (**A**,**B**) (SE). (**D**) Illustrative image of MeHA patch and HY 2% gel. **** *p* < 0.0001.

The 2% hyaluronic acid formulation and MeHa patches were enriched with ADNVs, and their release was evaluated by the protein concentration (Figure 2).

ADNVs’ release profile from the 2% hyaluronic acid formulation and MeHA patches were monitored by the quantification of the protein content in the reservoir (Figure 2D). Each trend, at both room and physiological temperature, is depicted in Figure 2A,B. The experiment underlined temperature’s pivotal role in ADNV release. Room temperature (RT) allowed a much slower and lower delivery than physiological temperature (37 °C). In fact, the cumulative release of ADNVs from MeHA patches, individuated by the areas beneath the curves, amounts to 853 μg and 1430 μg of proteins per mL at RT and 37 °C, respectively. The release from HY 2% gel is one order of magnitude lower compared to MeHA (RT, 182 μg; 37 °C, 440 μg) (Figure 2C). The gel promotes a steadier release at both temperatures. MeHA, on the other hand, produces a steeper and downward profile, especially at 37 °C.

### 3.3. MTT and Migration Assay

**Figure 3:**
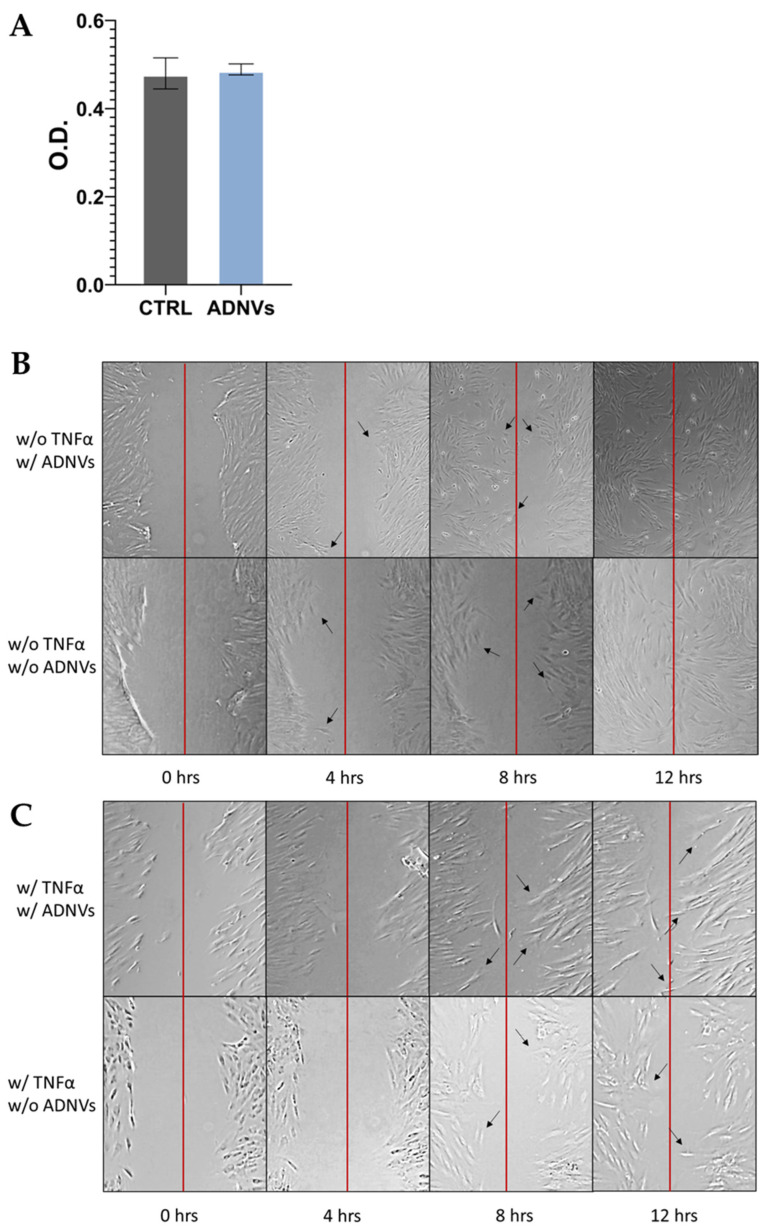
(**A**) MTT results for NCTC L929 cells grown in control medium (CTRL) and in ADNV-spiked medium (ADNVs); cell viability is expressed as absorbance. (**B**,**C**) Migration assay results are displayed as images taken in time, from 0 to 12 h (hours); the red line indicates the scratch’s middle, while black arrows point towards cells that have significantly moved since the previous image was taken.

The cytotoxicity of ADNVs was probed by MTT assay on NCTC L929 cells. The test resulted in no visible changes in cellular viability between the control and treated samples (Figure 3A). The migration progress was monitored for 12 h in fibroblasts either w/ or w/o TNFα. No significantly viable data were obtained by the experiment, only visual confirmation that the cell migration capability was not interfered with by ADNVs. In Figure 3B, cells w/o TNFα can be seen reaching the middle of the scratch (red line) after 8 h, while complete coverage of the empty space is reached at 12 h. In cells treated w/ TNFα (Figure 3C), the progression was much slower compared to cells w/o TNFα. However, there was no tangible difference among treated and non-treated samples.

### 3.4. Data Distribution of RNA Sequencing Data

Illumina-based sequencing was performed on ten independent samples corresponding to the whole transcriptome analysis of fibroblasts in inflamed and non-inflamed conditions (w/ or w/o TNFα), which were or were not further exposed to ADNVs (w/ or w/o ADNVs). The sequencing results were compared and analyzed, as described in Section 2.10.

**Figure 4:**
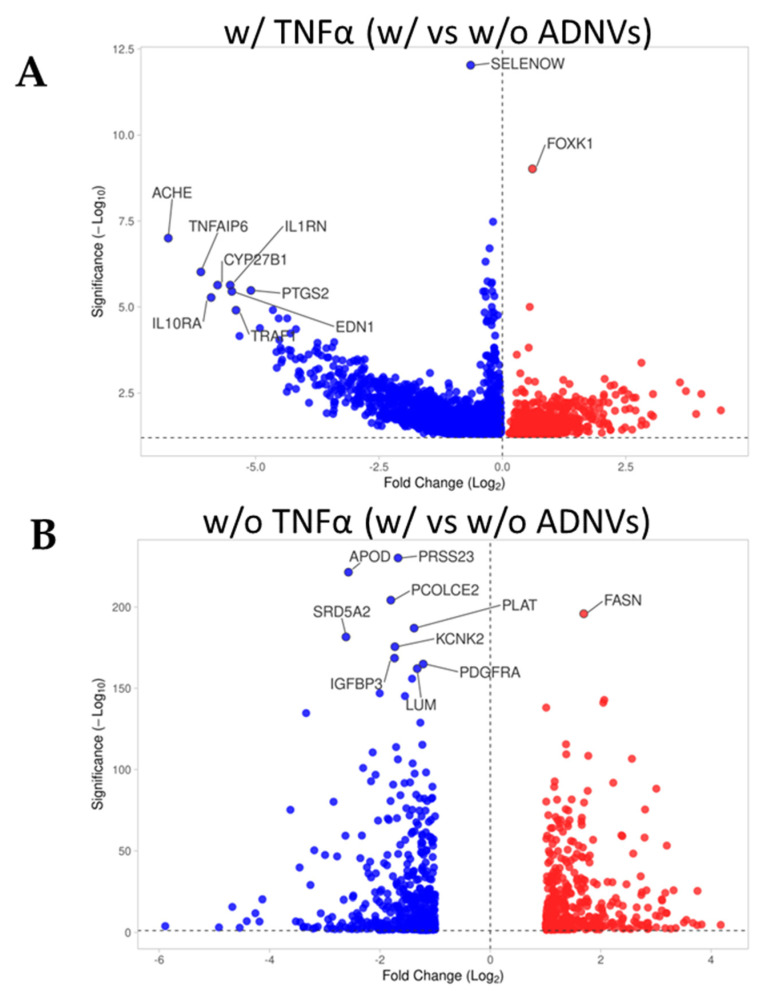
(**A**,**B**) Volcano plot of microarray outputs for primary dermal fibroblasts grown w/ TNFα (**A**) or w/o TNFα. Both graphs represent control (w/o ADNVs) vs treated (w/ ADNVs) conditions. Ten most relevant genes in terms of fold change and significance are reported.

By observing the data distribution of the two comparison datasets, a general understanding of differential gene expression induced by ADNVs treatment can be gained (Table 3). Firstly, ADNV treatment influenced cellular response through the up- and downregulation of several genes. Secondly, the inflammation status of cells conditioned the degree of the ADNV treatment’s effect. In fibroblasts w/ TNFα, 2792 differentially regulated genes (DRGs) were identified. Of these, 25.1% are upregulated and 74,9% are downregulated. On the other hand, fibroblasts w/o TNFα showed a difference of less than half DRGs between control conditions and ADNV-treated samples (876 DRGs, 44.8% upregulated and 55.1% downregulated). The difference between up- and downregulated gene distribution can be better visualized in Figure 4A,B, where most DRGs of each dataset are reported in relation to their fold change (FC) and *p*-value. The most significant DEGs are reported for each dataset, among the w/ TNFα genes that can be noticed are PTGS2 and IL1RN.

### 3.5. Pathway Enrichment Analysis of Inflamed Fibroblasts (w/ TNFα) Datasets

**Figure 5:**
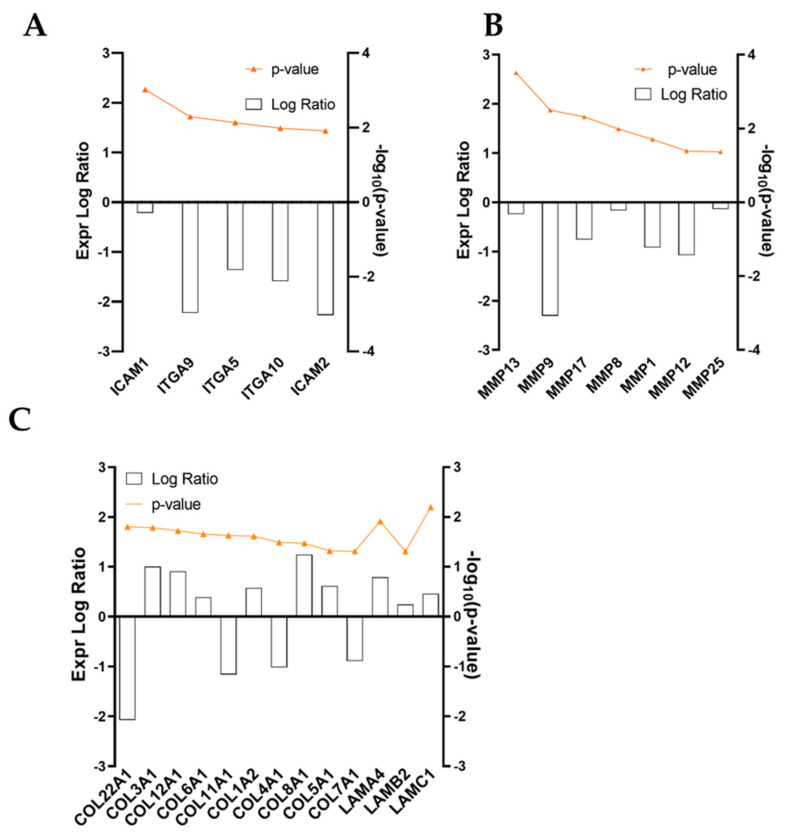
(**A**–**C**) Expression logarithmic ratios of differentially regulated genes from the w/ TNFα microarray involved in ECM maintenance. Expression is displayed as a bar, while significance, expressed as −log10(*p*-value), is reported in orange.

The high number of entries obtained from RNA-seq analysis was scanned up front to find molecules involved in inflammation and regeneration processes (Figure 5). During this scrutiny, several ECM-protein-related genes were identified as molecules significantly regulated by ADNV treatment. Collagen chains COL3A1, COL1A2, COL8A1 and COL6A1 were twice as expressed compared to control conditions, while COL4A1, COL5A1, COL7A1 and COL1A1 were significantly downregulated (Figure 5C). Laminin genes LAMC1 (Laminin Subunit Gamma 1), LAMA4 (Laminin Subunit Alpha 4) and LAMB2 (Laminin Subunit Beta 2) were also overly expressed by ADNVs (Figure 5C). On the other hand, ADNVs reduced, up to three times, the expression of integrins for cell–cell and cell–matrix adhesion, such as ITGA9, ITGA5 and ITGA10, and intercellular adhesion molecules ICAM1 and ICAM2, except for matrix adhesion molecules ECM2 (Extracellular Matrix Protein 2) (Figure 5A). Amidst ECM molding enzymes, several extracellular metalloproteinases were inhibited by ADNV treatment (Figure 5B). The downregulation of MMP1, MMP8 and MMP9 is relevant for their focal role in ECM degradation.

**Figure 6:**
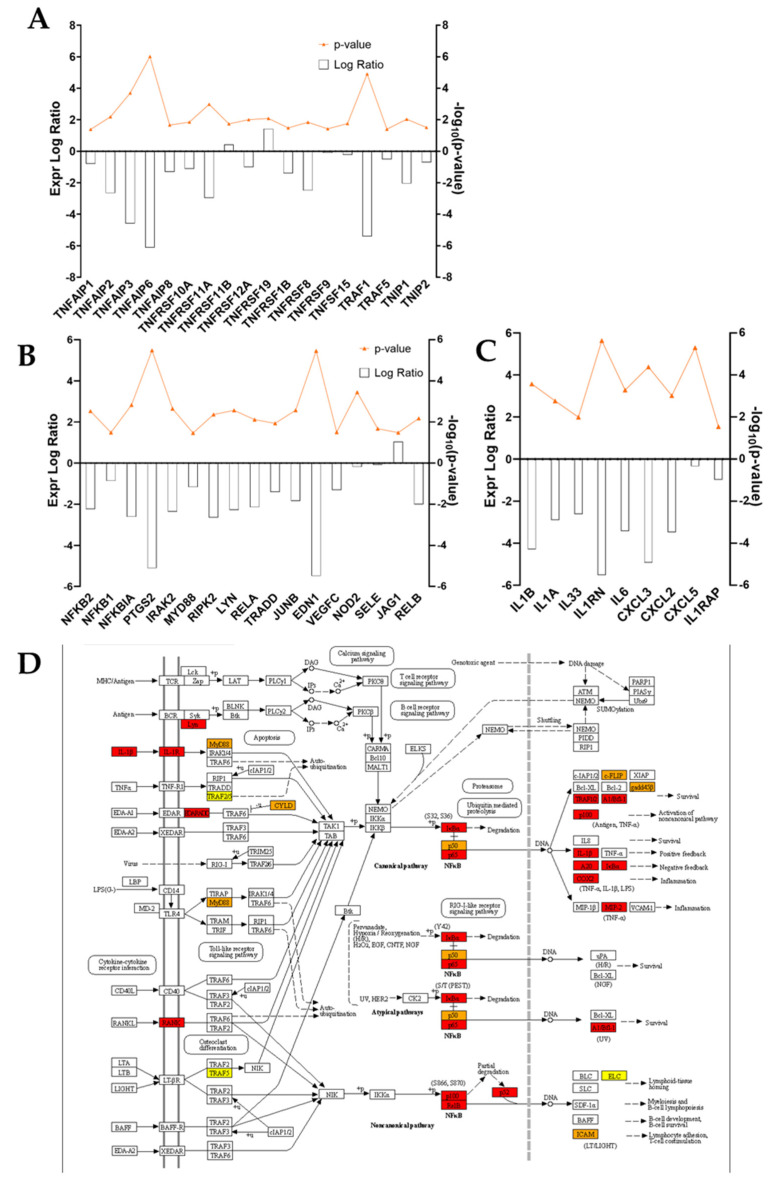
(**A**–**C**) Expression logarithmic ratios of differentially regulated genes from the w/ TNFα microarray involved in the inflammation response. Expression is displayed as a bar, while significance, expressed as −log10(*p*-value), is reported in orange. (**D**) KEGG pathway: IL-1b and NF-κB pathway, with color-coded protein slots corresponding to DEGs expression.

In addition to ECM components, a pool of pro- and anti-inflammatory markers was investigated. Figure 6C shows the expression ratio of differentially regulated Interleukins and chemokines of interest. The Interleukins of the IL-1 family—IL-1β, IL-1α and IL-33—are negatively regulated in ADNV-treated samples, together with Interleukin 1 receptor (IL1RN). Among Interleukins of other families, solely Interleukin 6 (IL-6) displayed a significant change in expression (Expr. Log Ratio = −3.46), while Interleukin 8, 4, 10 and 13 families did not experience any shifts. For what concerned chemokines, the RNA-seq dataset provided information on the expression of CXCL3, CXCL2 and CXCL5, all less expressed in ADNV-treated samples. Another inflammation marker is Tumor Necrosis Factor, jointly with TNF-related proteins. ADNVs negatively regulate several of these factors, enzymes and receptors, as listed in Figure 6A, with only the exception of TNFRSF19 and TNFRSF11B. Another panel shows genes involved in the NF-κB pathway, all of which are downregulated except for Jagged Canonical Notch Ligand 1 (JAG1) (Figure 6B). Among them, there are Nuclear Factor Kappa B Subunit 1 and Subunit 2 (NFKB1 and NFKB2), NFKB Inhibitor Alpha (NFKBIA), MYD88 Innate Immune Signal Transduction Adaptor (MYD88), Prostaglandin-Endoperoxide Synthase 2 (PTGS2 or COX2) and Interleukin 1 Receptor Associated Kinase 2 (IRAK2). All these pieces of information are condensed in the scheme of Figure 6D, portraying both canonical and non-canonical NF-κB signaling pathways. From IL-1b signaling to TNFα-induced expression, the KEGG pathway chart was combined with each expression log ratio value to allow the reader to better visualize the dynamics among DEGs involved.

**Figure 7:**
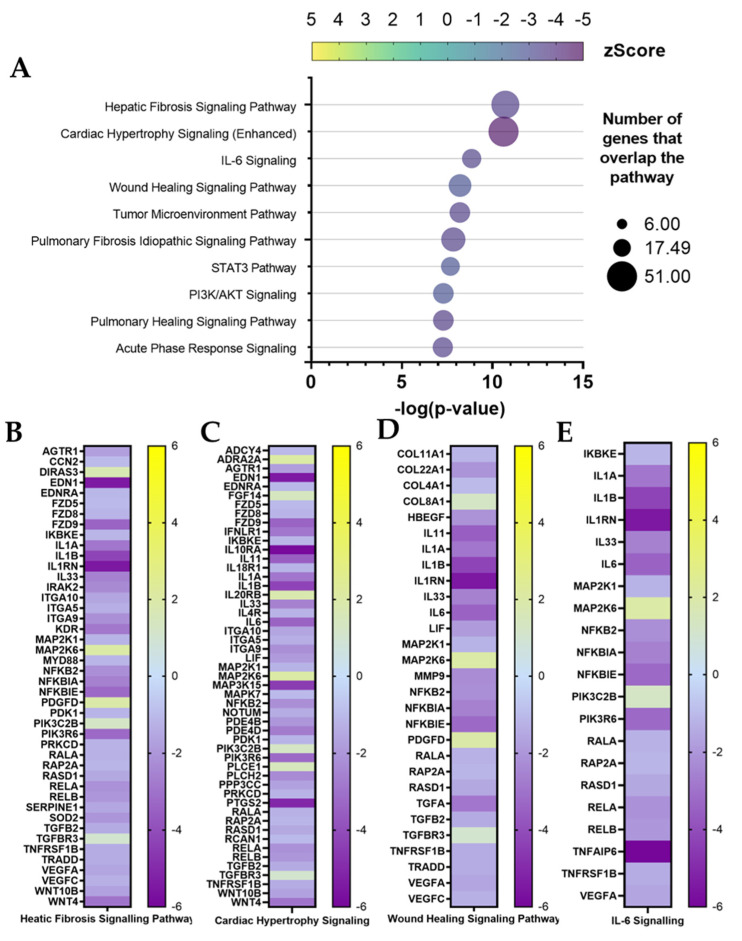
Canonical pathway analysis by IPA for w/ TNFα. (**A**) Ten first entries. (**B**–**E**) Genes involved in the first four pathways with expression log ratio values.

Inflamed fibroblast (w/ TNFα) RNA-seq output was further analyzed with Ingenuity Pathway Analysis (IPA) software. The IPA gene ontology algorithm classifies canonical pathways based on their statistical relevance (*p*-value) and activation status (z-Score) by identifying each DEG’s role in specific biological pathways. Among the ten higher ranking canonical pathways (Figure 7A), the top two are related to illnesses involving pro-inflammatory activity and ECM composition. First, is the hepatic fibrosis signaling pathway (−log(*p*-value) = 10.7; z-Score = −3.57), and next is the cardiac hypertrophy signaling (−log(*p*-value) = 10.6; z-Score = −4.72). Both pathways scored negatively. Therefore, IPA’s prediction considers them inactivated by ADNV treatment compared to control conditions. Each of these pathways involved more than 50 DEGs, which expression is shown in Figure 7B,C.

Among them that can be noticed are previously mentioned proteins involved in ECM maintenance (ITGA5, ITGA9 and ITGA10) and inflammation (IL1B, IL1A, IL1RN, IL33, IL-6, IRAK2, MYD88, NFKB2, RELA, RELB, SOD and PTGS2). Several of the other higher-ranking pathways are involved in the process of inflammation: IL-6 signaling pathway (−log(*p*-value) = 8.84; z-Score = −3.44), wound healing signaling pathway (−log(*p*-value) = 8.2; z-Score = −2.78) and, partially, STAT3 pathways (−log(*p*-value) = 7.67; z-Score = −2.8). Compared to hepatic fibrosis and cardiac hypertrophy pathways, fewer DEGs are part of IL-6 and wound healing signaling networks (Figure 7D,E). The wound healing pathway included gene coding for ECM proteins (COL8A1, COL4A1, COL22A1 and COL11A1), metalloproteinase 9 and several Interleukins (IL1B, IL1A, IL33 and IL6). Several of these molecules are involved in IL-6 signaling as well, while certain DEGs, such as Interleukin 1β (from IL1B) and Interleukin 1 receptor (IL1RN), are common to all four canonical pathways. Other less significant (−log(*p*-value) < 6) pathways are mentioned when concerning the inflammation and regeneration process (Table S1). Interleukin 1 signaling is inactivated by ADNVs (z-Score = −2.3), as well as Interleukin 8 signaling (−2.8), Interleukin 3 signaling (z-Score = −2.1), NF-κB signaling (z-Score = −2.1), iNOS signaling (z-Score = −2.8) and the inflammasome pathway (z-Score = −2).

**Figure 8:**
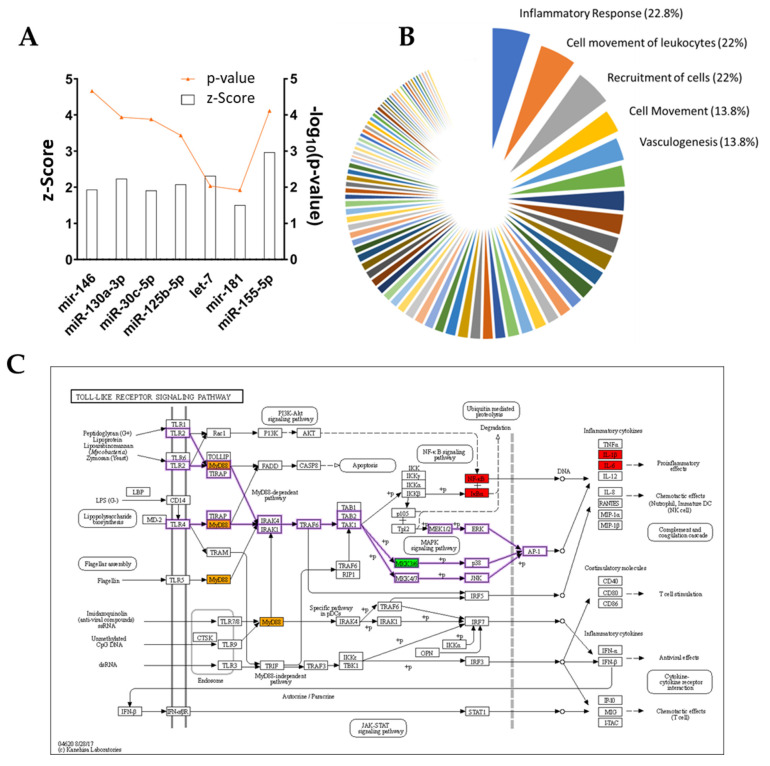
(**A**) Upstream miRNA regulators for w/ TNFα IPA analysis, z-Score and *p*-value are shown. (**B**) IPA regulator’s ranked by influenced functions, shown as percentage of the total regulator effects. (**C**) KEGG pathway: Toll-like receptors signaling pathway, with color-coded protein slots corresponding to DEGs expression.

Another of IPA’s features is the prediction of possible regulatory molecules, the activation or inhibition of which could explain DEGs output. In this dataset, IPA identifies TNFA (−log(*p*-value) = 37.2; z-Score = −8.9) and IL1B (−log(*p*-value) = 33.7; z-Score = −8) as the most significant regulatory molecules (Table 4). Among the top six regulators are also the cytokines IL33 (−log(*p*-value) = 19.2; z-Score = −4.6), IL4 (−log(*p*-value) = 17.9; z-Score = −5.9), IFNG (−log(*p*-value) = 15.7; z-Score = −6), the NFkB complex (−log(*p*-value) = 19; z-Score = −7), TGFB1 (−log(*p*-value) = 18.9; z-Score = −4.1) and NR3C1 (−log(*p*-value) = 19; z-Score = 2.2). All the aforementioned regulators are inhibited by ADNV treatment, except for NR3C1. Few miRNAs are mentioned in the complete list of predicted upstream regulators. Among them, there are miR125b-5p (z-Score = 2.7), let-7 (z-Score = 2.31), miR-155-5p (z-Score = 2.9), mir-146 (z-Score = 1.9) and miR-181 (z-Score = 1.5) (Figure 8A).

In fibroblasts grown w/ TNFα, ADNV treatment induces a decrease in the inflammatory response and inflammatory diseases, as shown in Table 5 “Diseases and Functions”.

Lastly, IPA discovered potential novel regulatory networks and causal relationships associated with RNA-seq data. The networks tied i) ADNVs treatment, ii) master regulators and iii) their effects in terms of the activation/deactivation of canonical pathways and other diseases and functions. All predictions are ranked by consistency score (CS) and have been clustered according to their downstream functions (Figure 8B). Most targeted is the category “Inflammatory Response” (22.8%), followed by “Cell movement of Leukocytes” (22%) and “Cell Recruitment” (22%). In the top 20, we also find “Activation, Cell movement and Adhesion of Leucocytes”. Among all predicted networks, one regulated by Toll-like Receptor 4 (TRL4) is the second highest ranking in terms of CS score (consistency score, 3.5; total nodes, 22; and ranking position, 2) (Table 6).

This network comprises 20 downstream DEGs (ADM, CSF3, CXCL2, CXCL3, EDN1, IL1A, IL1B, IL6, INHBA, MMP9, MYD88, NFKB2, NFKBIA, NFKBIZ, PTGES, PTGS2, RELA, RIPK2, TICAM1 and TNFAIP3) and connects these to “Inflammatory response” moderation. A representation of all molecules involved in the Toll-like receptors pathway can be observed in Figure 8C, where each molecule of the KEGG pathway is color-coded depending on the expression ratio value for the corresponding gene. Other significant networks with high CS are listed in Table 6, including one regulated by IL1RN targeting leukopoiesis, one regulated by TRL4 targeting the cell movement of leukocytes, a PTGS-regulated network for cell expansion, a TNFSF14-regulated network for the inflammatory response and an MYD88-regulated network for the adhesion of mononuclear leukocytes.

### 3.6. IPA Analysis of Non-Inflamed Fibroblasts Datasets

**Figure 9:**
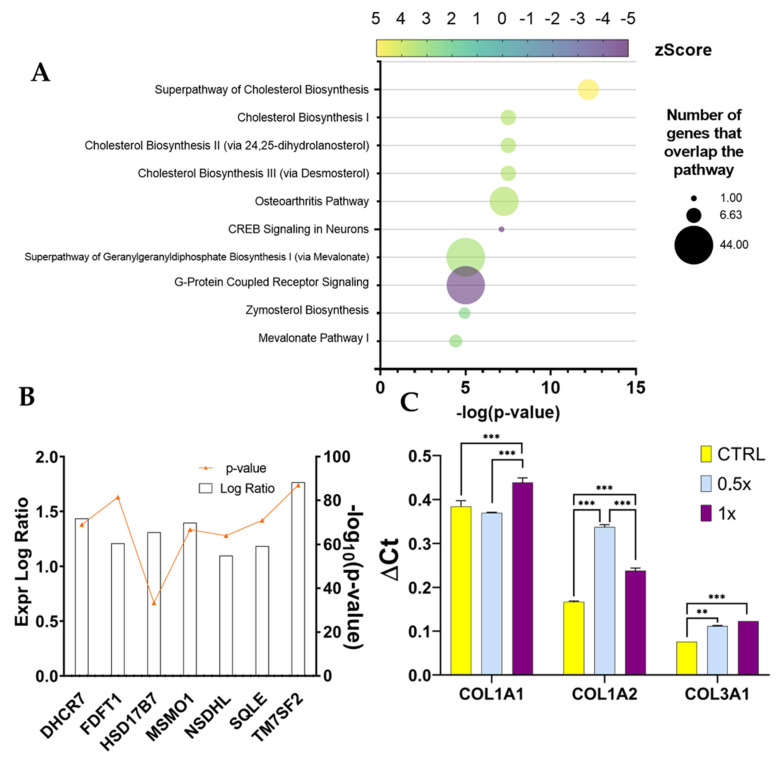
(**A**) Canonical pathway analysis by IPA for w/o TNFα. (**A**) Ten first entries. (**B**) Gene expression of genes involved in cholesterol biosynthesis (w/o TNFα dataset); expression is displayed as a bar, while significance, expressed as −log10(*p*-value), is reported in orange. (**C**) RT-qPCR results for gene expression in w/ TNFα fibroblasts after 1× and 0.5× ADNV treatment (** *p* < 0.01, *** *p* < 0.001).

The comparison between fibroblasts grown with the control medium (w/o TNFα), w/ or w/o ADNVs was also fed to IPA software. The most significant canonical pathways discovered by IPA concern cholesterol biosynthesis: the super pathway of cholesterol biosynthesis (−log(*p*-value) = 12.2; z-Score = 3.6), cholesterol biosynthesis I (−log(*p*-value) = 7.5; z-Score = 2.6), II (via 24,25-dihydro-lanosterol) (−log(*p*-value) = 7.5; z-Score = 2.6) and III (via Desmosterol) (−log(*p*-value) = 7.5; z-Score = 2.6) (Figure 9A). All have positive z-Scores, indicating activation in ADNV-treated samples. In the small number of molecules of the DRGs list that matches cholesterol biosynthesis, seven can be identified as the common core: DHCR7, FDFT1, HSD17B7, MSMO1, NSDHL, SQLE and TM7SF2 (Figure 9B). All aforementioned genes were upregulated by the addition of ADNVs to the medium.

Several upstream regulators were also identified by IPA in this dataset (Table 7). The first, most relevant regulators are Mitogen-Activated Protein Kinase Kinase 5 (MAP2K5) (−log(*p*-value) = 15.1; z-Score = 4.7) and Mitogen-Activated Protein Kinase 7 (MAPK7) (−log(*p*-value) = 12.8; z-Score = 3.7), both activated in ADNV-treated samples. However, the z-Scores of diseases and functions do not bypass the established cut-off (z-Score < −2 or z-Score > +2).

A total of 22 multi-regulated networks were discovered by IPA. All entries are unique in terms of targeted functions. The higher ranking network (Table 8) is composed of 39 total nodes, of which 8 are regulators (APEX1, CEBPA, CYP19A1, EGFR, GHR, IDO1, NEDD9 and WBP2), 27 are target molecules (AGT, CCL2, COL5A2, CSF2, GDF15, HGF, HMGCR, IGF1, IL33, IL6, ITGAX, LEP, LIPG, MMP14, MMP3, NR4A1, PLAT, PLIN2, PTGES, PTGS2, PTX3, SCARB1, SCD, SLC7A11, SPP1, THBD and TNFSF10) and 4 are diseases and functions (the apoptosis of renal tubular epithelial cells, cerebrovascular dysfunction, the occlusion of blood vessel and vaso-occlusion).

### 3.7. RT-qPCR

After RNA sequencing analysis, three biological markers (COL3A1, COL1A1 and COL1A2) were chosen to assess the influence of ADNV concentration on the physiological effect of inflamed fibroblasts. RT-qPCR results are displayed as ΔCt values in Figure 9C. The ADNV-to-effect trend of expression is linear in the case of COL3A1, with significant changes between each sample. However, the expression of COL1A1 does not increase after 0.5× ADNV exposure (100 μg/mL) but only after full exposure (200 μg/mL). Both COL3A1 and COL1A2 chain expression increased in a dose-dependent manner. While COL3A1 expression increment is linear, COL1A2 exhibits a biphasic trend: a lower dose response (100 μg/mL) is more pronounced compared to a higher dose response (200 μg/mL). These results line up with the hormesis model for dosage–response curves.

### 3.8. Oxidative Stress Evalutation

**Figure 10:**
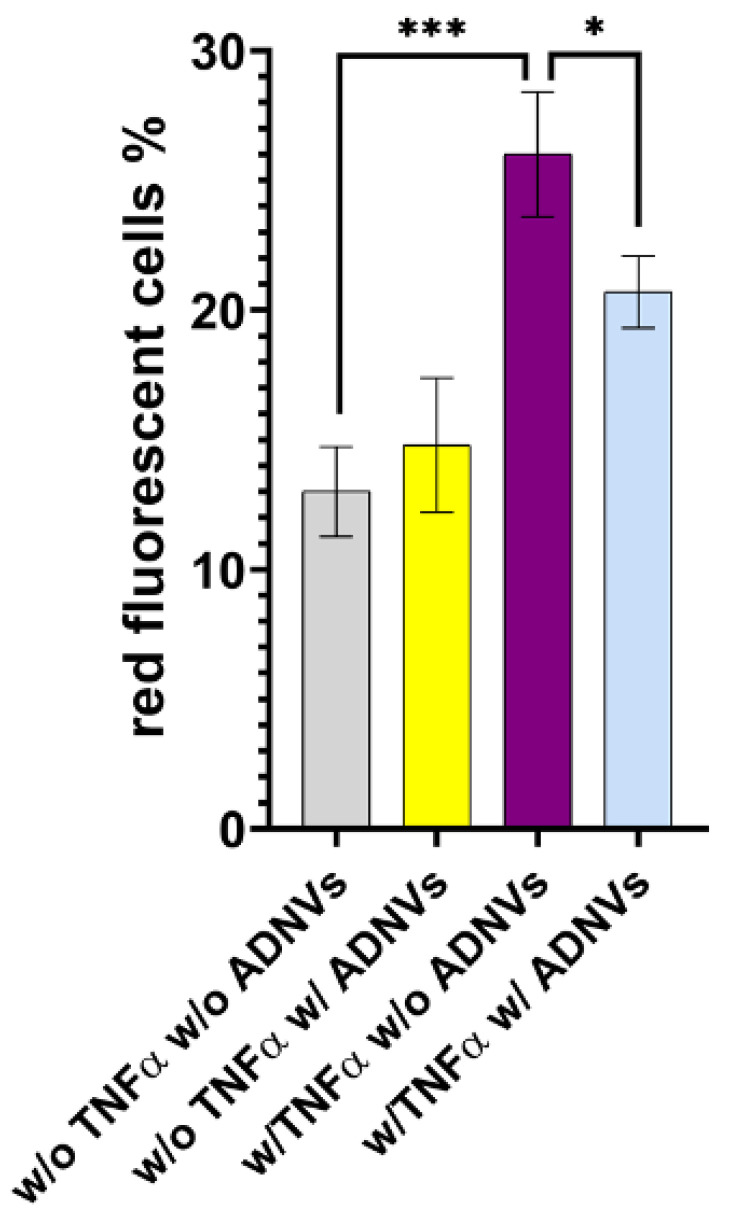
Mitochondrial stress under all conditions is expressed as percentage of cells with above mitochondrial ROS production. * *p* < 0.05, *** *p* < 0.001.

Mitochondrial ROS levels were assayed with MitoSOX^TM^ fluorescent probe (Table 9). Over a total of 1.38 × 10^6^ cells cultured w/o TNFα and w/o ADNVs, 13% exhibited mitochondrial stress and ROS production significantly lower compared to cells treated w/ TNFα (1.34 × 10^6^, 26% red positive). As expected, cells treated w/ TNFα exhibited a higher level of stress compared to cells w/o TNFα condition. Interestingly, treatment w/ ADNVs decreased the level of ROS production in inflamed cells (w/ TNFα) by 5%, while treatment with ADNVs in non-inflamed cells did not produce any quantifiable variation in mitochondrial ROS (Figure 10).

## 4. Discussion

Vegetable-derived nanovesicles are a novelty in the field of regenerative medicine. Nonetheless, much is yet to be discovered on the changes they induce in human cells. In this study, we focused on apple-derived nanovesicles as bioactive principles for cosmetic applications and their role in skin rejuvenation. RNA sequencing was used to comprehensively characterize the effect of ADNVs on human primary dermal fibroblasts, which are first responders during tissue regeneration.

The Golden delicious apple variety was chosen for its popularity among producers and consumers. Here, ADNVs were physically characterized through TRPS and electron microscopy imaging. The findings were regarded comparable to previously published studies [32,34,50,51]. The dimension and shape of ADNVs were parallel to other vegetable-derived nanovesicles and the ones already described for apples. Cytotoxicity assays were employed to provide data on ADNV harmfulness to human cells (Figure 3A). It was established that ADNVs are not harmful to human cells by the results of cell vitality assay on both inflamed and non-inflamed fibroblasts, as per previously published data [44].

After establishing their safety, investigators explored ADNVs’ role in skin preservation. Two main events associated with intrinsic skin ageing are the decrease in the replicative ability of cells (cellular senescence) and the increased degradation of the ECM due to ROS activity [8,9]. Fibroblasts in the dermis are the greatest producers of collagen and other dermal proteins that constitute the ECM. Therefore, fibroblast migration and ECM production are indispensable for tissue regeneration and healing. This study established that ADNVs do not affect fibroblast migration significantly, both in a situation of inflammation and non-inflammation (Figure 3B,C). However, RNA sequencing analysis has helped to describe the metabolic changes induced by ADNVs in fibroblasts w/ or w/o the influence of pro-inflammatory factors. Here, fibroblasts have responded more strongly to ADNVs when previously subjected to pro-inflammatory treatment (w/ TNFα) (Figure 4). In such conditions, fibroblasts grown in an ADNV-spiked medium responded through the downregulation of genes involved in IL-1β signaling and TNFα signaling pathways (Figure 6A–C). An abnormal expression or activity of pro-inflammatory cytokines and factors is expected in non-healing wounds, as described by several authors through time [52,53]. Thus, the inactivation of IL-1β and TNFα signaling pathways is evidence of lower inflammation in ADNV-treated fibroblasts [54]. In physiological conditions, IL-1β and TNFα promote the activation of the canonical NF-κB signaling pathway: NF-κB transcription factor, translocating into the nucleus, increments positive feedback to IL-1, signaling the transcription of pro-inflammatory cytokines (IL-8 and IL-6) and chemokines (CXCL5 and CXCL4) [9,55,56]. In accordance with this premise, proteins associated with NF-κB signaling were decreased by ADNV treatment (Figure 6D). Prostaglandin-Endoperoxide Synthase 2 (PTGS2) is a crucial gene in prostaglandin E2 synthesis. One mechanism of skin erythema activation by ROS is through increased prostaglandin E2 levels [10]. Thus, the downregulation of PTGS2 contributes to the overall picture of ADNVs’ anti-ageing effect. This is also confirmed by the diseases and functions ranking of IPA, which indicates an overall decrease in inflammatory response and inflammatory diseases (Table 5).

Unsurprisingly, IL-1β and TNFα have been pinpointed as deactivated regulators of an ADNV-induced response in inflamed fibroblasts by IPA (Table 4). A few miRNAs were also included in the list of possible upstream regulators, such as miR125b-5p and mir-146 family (Figure 8A). As a previous study uncovered [44], these miRNAs are key regulators for anti-inflammatory behavior in THP-1-derived macrophages treated with ADNVs. Their positive regulation in both fibroblasts and macrophages adds another layer of complexity to the extent that ADNVs can reduce pro-inflammatory signals in human cells. Moreover, IPA software recognized a prominent TLR4-regulated network in fibroblasts’ ADNV response (Table 6). TRL4 belongs to the Toll-like receptors family and acts via MYD88, TIRAP and TRAF6 (factors involved in the IL-1 receptor and TNFα signaling pathways) to activate NF-κB signaling and mediate the innate immune response (Figure 8C) [57]. The network’s identified targets are all the DEGs involved in the inflammatory response (MYD88, PTGS2, IL1B and IL1A) as it is its final downstream function regulation. Interestingly, miRNA 146a and 125a are also involved in the negative regulation of TLR4, as described by several previous investigations [58,59,60]. As such, the TLR4-regulated cellular response to ADNVs was considered one possible response mechanism of macrophage-derived THP-1 to ADNVs [44].

In summary, all evidence points to a clear mechanism of action provoked by ADNVs in fibroblasts w/ TNFα, resulting in the negative regulation of NF-κB pathways and, overall, a suppressed pro-inflammatory response. Given the causal relationship between ROS-induced skin damage and inflammation, we can deduce that ADNV-induced reduction in inflammatory signaling could counteract the effects of oxidative-stress-induced ageing. Other predicted networks reinforce the connection between ADNVs action and the anti-inflammatory effect (Figure 8B). Several of them target “Cell movement”, which plays a crucial role in regeneration; wound healing is given by a cells’ capacity to migrate and produce ECM, and the cells of the immune systems are required to migrate to the site of action. As determined by scratch assay, migration progress was not delayed by ADNV treatment (both w/ and w/o TNFα). However, to establish a quantitative significant difference between treated and control, a more extended study needs to be conducted.

Aside from molding the inflammation response, ADNVs’ effect has been surveyed concerning the changes in ECM production by fibroblasts w/ TNFα. Collagen fibers are structural proteins that play critical roles in providing tensile strength to the skin [17]. However, collagen is a frequent target of modifications by ROS due to its slow turnover rates [61]. For this reason, monitoring its production rate is essential for assessing the ability of fibroblasts to supply new matrices. Type I collagen is most abundant in the dermal ECM associated with type III collagen. Type IV is also involved in skin health, as it is found in the basal lamina. This investigation observed an ADNV-induced increase in collagen type I α2 and collagen type III chain α1, contrasted by a decrease in collagen type I α1 chain and collagen type IV α1 chain (Figure 5C). In addition to an accelerated matrix turnover, ADNVs promote mitochondrial fitness in inflamed fibroblasts by decreasing oxidative stress and ROS production (Figure 10). These preliminary data indicate the role of ADNVs in the preservation and maintenance of the ECM.

Under stressful conditions, activated NF-κB in dermal fibroblasts also represses collagen synthesis. This is achieved by increasing the MMP/TIMP balance [8]. MMPs are extracellular endopeptidases that differ greatly in substrate specificity and, thus, are collectively capable of breaking down several components of the ECM [62]. Thus, NF-κB signaling plays a well-established and essential role in ECM maintenance and, consequentially, in photoaging and intrinsic ageing. Indeed, MMP1, MMP8 and MMP9 are downregulated by ADNVs (Figure 5B). Metalloproteinase 1 and 8 both target interstitial collagen type I, II and III, while Metalloproteinase 9 is specialized in collagen type IV and V. Thus, together they would be able to break down and remodel the ECM with significant changes. Furthermore, MMPs have a part in other pathophysiological processes; particularly, MMP9 is considered a cancer biomarker [63,64].

In all, it is possible to say that the repression of the NF-κB pathway, in addition to the upregulation of collagen chains, laminin chains and the downregulation of pivotal MMPs, poses positive premises for ADNV treatment to induce ECM restoration. Given the centrality of its role, collagen synthesis was chosen as a marker to establish a correlation between quantity and effect in ADNV treatment. While COL3A1 expression is linearly dose-dependent, COL1A2 expression tendency is in accordance with the hormetic model. The lower dosage (100 μg/mL) stimulates COL1A2 expression in a stronger manner compared to higher dosage (200 μg/mL). However, higher dosage still stimulates a significant response compared to control conditions. Thus, collagen chain I a2 could be one of the most reliable markers for ADNV activity moving forward.

Another consideration can be made starting from RNA-seq analysis data. The inactivation of canonical pathways for fibrosis-related pathologies could indicate a diminished fibrotic healing mechanism by ADNV treatment (Figure 7A–C). ICAM1 is considered an indicator of fibrosis-inducing fibroblasts [65] as it can interact with integrins presented by infiltrated leukocytes. The interactions result in the activation of both fibroblasts and leukocytes, increasing the fibrotic response and cytokine IL-6 and IL-8 production. In this work, ICAM1, IL-6 and IL-8 were found downregulated by ADNVs in fibroblasts w/ TNFα, which could partially explain the results of the canonical pathway.

As already mentioned, fibroblasts grown in normal conditions (w/o TNFα) did not react as strongly to ADNVs. The results point to an increase in cholesterol biosynthesis tied to the expression of a handful of genes coding for the expression of cholesterol biosynthesis enzymes (Figure 9A). In the literature, the stimulation of cholesterol biosynthesis in dermal fibroblasts is loosely associated with bio-protection. An in vitro study by Chee et al. (2021) on naked mole-rat-derived cells linked cholesterol metabolism inhibition to increased oxidative stress [66]. However, data are scarce on cholesterol’s role in skin-derived fibroblasts. The master regulator identified by IPA, MAP2K5 (Table 7), is also involved in lipid metabolism regulation, together with ERK5 [67]. MAP2K5 activates MAPK7 that, in combination with MMP9, is associated with positive tumor-inducing signaling in cancer patients [63,68]. However, in healthy cells, MAPK2K5 is involved in several cellular processes, such as proliferation, differentiation, transcription regulation and development. IPA has also predicted the inhibition of WNT3A, involved in the canonical WNT signaling pathway that results in the activation of transcription factors of the TCF/LEF family. Overall, significant data are scarce in this dataset (w/o TNFα), and as such, the authors were not able to determine a significant and valid mechanism of function of ADNVs in this setting.

Lastly, this work found it appropriate to investigate ADNVs’ applicability in cosmetic formulations and medical dressing. Hyaluronic acid is a natural polysaccharide, which has been used in various forms and with numerous functionalization [69,70,71,72,73]. From topical applications to drug delivery, it has been adopted for biomedical and cosmetic solutions. Alone, it has demonstrated pharmacological activity against inflammation [74] and promising results in anti-ageing [75]. In the right formulation, it can easily encapsulate nanometric particles, and it is widely used for controlled drug release [46,76]. Therefore, HA was chosen to be combined with ADNVs to focus on and increase each other’s anti-inflammatory activities. In both hydrogel (HY 2%) and mesh form (MeHA patches), ADNVs were successfully encapsulated and released (Figure 2A–D). Therefore, they are both valid alternatives, although they differ in release rates. In conclusion, this solution can bring ADNVs to the site where tissue regeneration is needed to maximize their potential.

## 5. Conclusions

The skin is a barrier of the body, towards which care needs to be taken throughout our lives. Rejuvenating and regenerative therapies often involve novel active principles that tackle dermal inflammation and promote ECM synthesis. This study concluded that nanovesicles isolated from the apple variety Golden delicious exhibited anti-inflammatory properties in primary dermal fibroblasts. Fibroblasts that were already in a state of inflammation responded more significantly than the ones in a non-inflamed state. Although much needs to be discovered about the ADNV mode of action, it seemed to involve the downregulation of the NF-κB pathway through the dimming of TLR4-induced signals. The results further indicated a pattern change in collagen and laminin synthesis. Particularly, ADNVs induced a higher production of COL1A2. On the other hand, the effect of ADNVs on fibroblasts grown in normal conditions is to increase cholesterol biosynthesis. This aspect needs further exploration, as how this effect is achieved is still unclear. Moreover, this study found that hyaluronic-acid-based biomaterials are optimal candidates for ADNV application. The double form of gel and patches increases the potential uses of ADNVs, both in cosmetics and regenerative medicine. However, epidermal permeability in ADNVs will need to be addressed by future research by identifying suitable carriers and supports for ADNVs’ topical delivery to the derma. Overall, ADNVs exhibited an unveiled potential for several applications. A more in-depth characterization of their effect on other varieties of cell types is necessary, however, to appreciate the extent of ADNVs’ possible uses.

## Figures and Tables

**Table 1:** Primer sequences for RT-PCR.

Gene	FOR	REV
COL1A1	TGGTGACAAGGGTGAAAGTG	GCATCGCCTTTAGCACCAG
COL1A2	CTTGGTGCTCCTGGTATTCTG	TGACTCCAGGACTACCCAC
COL3A1	GGAAGAGATGGAAACCCTGGA	CACTTTCTCCTCTGTCACCAC
ACTB1 ^1^	GCATCCACGAAACTACCTTCAACTC	CTTGATCTTCATTGTGCTGGGTG

^1^ Housekeeping gene (HKG).

**Table 2:** TRPS analysis data.

Sample	Mean Diameter (nm)	Mode Diameter (nm)	Raw Conc (/mL)	Particle Count	Particle Rate (/mL)
ADNVs_1_p20	124	117	5.1 × 10^9^	849	337.6
ADNVs_1_p10	115	104	5.1 × 10^9^	633	290
ADNVs_2_p20	120	108	5.8 × 10^9^	761	252.3
ADNVs_2_p10	112	106	5.8 × 10^9^	664	201.4
ADNVs_3_p20	130	110	2.5 × 10^11^	1130	2830
ADNVs_3_p10	115	104	2.1 × 10^11^	633	290

**Table 3:** General data distribution of microarray analysis and comparison.

	w/ TNFα (w/ vs w/o ADNVs)	w/o TNFα (w/ vs w/o ADNVs)
Total DEGs	2.792	876
Upregulated	701	393
Downregulated	2.091	483

**Table 4:** Upstream regulator list by IPA for w/ TNFα dataset.

Upstream Regulator	Expr Log Ratio	Molecule Type	Predicted Activation State	Activation z-Score	−log(*p*-Value)
TNF	−0.11	Cytokine	Inhibited	−8.97	37.22
IL1B	−4.29	Cytokine	Inhibited	−8.03	33.72
HIF1A	−0.73	Transcription regulator	Inhibited	−5.95	26.64
IL33	−2.61	Cytokine	Inhibited	−4.58	19.20
NR3C1	−0.16	Ligand-dependent nuclear receptor	Activated	2.19	19.09
NFkB (complex)		Complex	Inhibited	−7.07	19.03

**Table 5:** Diseases and Functions list by IPA for w/ TNFα dataset.

Categories	Annotation	−log(*p*-Value)	Predicted Activation State	Activation z-Score
Inflammatory response	Inflammatory response	6.34 × 10−^14^	Decreased	−4.90
Inflammatory response and neurological disease	Inflammation of central nervous system	2.28 × 10−^11^	Decreased	−2.55
Inflammatory disease, inflammatory response, neurological disease, organismal injury and abnormalities	Encephalitis	3.18 × 10−^10^	Decreased	−2.58

**Table 6:** Regulators effect: list of predicted networks by IPA for w/ TNFα dataset.

ID	Consistency Score	Node Total	Regulators	Target Total	Diseases and Functions
2	3.57	22	TLR4	20	Inflammatory response
3	3.47	12	IL1RN	10	Leukopoiesis
6	3.47	26	TLR4	24	Cell movement of leukocytes
18	3.33	11	PTGS2	9	Expansion of cells
45	3.16	12	COL18A1	10	Angiogenesis
46	3.16	12	FGF2	10	Fatty acid metabolism
47	3.16	12	IL1B	10	Adhesion of mononuclear leukocytes
462	2.44	8	let-7	6	Activation of cells
718	2.23	7	TNFSF14	5	Inflammatory response
995	2	6	MYD88	4	Adhesion of mononuclear leukocytes

**Table 7:** Upstream Regulator list by IPA for w/o TNFα dataset.

Upstream Regulator	Expr Log Ratio	Molecule Type	Predicted Activation State	Activation z-Score	−log(*p*-Value)
MAP2K5	−0.12	Kinase	Activated	4.71	15.15
CREB1	−0.17	Transcription regulator	Activated	2.35	13.02
MAPK7	−0.07	Kinase	Activated	4.11	12.82
SCAP	0.08	Other	Activated	4.38	12.50
SREBF1	0.24	Transcription regulator	Activated	5.27	10.79
INSIG1	1.23	Other	Inhibited	-4.36	10.43

**Table 8:** Regulators’ effect: list of predicted networks by IPA for w/ TNFα dataset.

ID	Consistency Score	Node Total	Regulators	Target Total	Diseases and Functions
1	10	39	APEX1, CEBPA, CYP19A1, EGFR, GHR, IDO1, NEDD9 and WBP2	27	Apoptosis of renal tubular epithelial cells, cerebrovascular dysfunction, occlusion of blood vessel and vaso-occlusion

**Table 9:** Mitochondrial ROS quantification through MitoSOX^TM^ imaging.

Sample	Concentration (Cell/mL)	% Cells
W/o TNFα w/o ADNVs	1.38 × 10^6^	13
W/o TNFα w/ ADNVs	1.12 × 10^6^	16
W/ TNFα w/o ADNVs	1.34 × 10^6^	26
W/ TNFα w/ ADNVs	1.39 × 10^6^	21

## Data Availability

Not applicable.

## Supplementary Materials

The following supporting information can be downloaded at: https://www.mdpi.com/article/10.3390/cells11243950/s1, Table S1: Other less significant [−log(*p*-value) < 6] canonical pathways for w/ TNFα dataset.
