# Supplementary material for: Apple Derived Exosomes Improve Collagen Type I Production and Decrease MMPs during Aging of the Skin through Downregulation of the NF-κB Pathway as Mode of Action
Source: Cells. 2022 Dec 7;11(24):3950. doi: 10.3390/cells11243950 (PMC9776931; doi:10.3390/cells11243950)
Supplement: Supplementary file 1 [file cells-11-03950-s001.zip › cells-2047126-supplementary.pdf]

Table S1. Other less significant [-log(p-value) < 6] canonical pathways for w/ TNFα dataset.

Canonical Ptw	$-\log_{10}$ (*p*-value)	z-Score	Molecules
Inflammasome	2.42	−2	IL1B,MYD88,NFKB2,PANX1
NF-κB Signaling	1.59	−2.11	BMP2,BMPR1B,CARD10,FCER1G,IL1A,IL1B,IL1RN,IL33,KDR,MAP2K6,MYD88,NFKB2,NFKBIA,NFBIE,PIK3C2B,PIK3R6,RALA,RAP2A,RASD1,RELA,RELB,TGFA,TGFBR3,TNFAIP3,TNFRSF11A,TNFRSF1B,TNIP1,TRADD
IL-3 Signaling	2.92	−2.12	INPP5D,MAP2K1,PIK3C2B,PIK3R6,PPP3CC,PRKCD,RALA,RAP2A,RASD1
IL-1 Signaling	3.47	−2.33	ADCY4,IKBKE,IL1A,IRAK2,MAP2K6,MYD88,NFKB2,NFKBIA,NFKBIE,RELA,RELB
iNOS Signaling	4.69	−2.82	IKBKE,IRAK2,JAK3,MYD88,NFKB2,NFKBIA,NFKBIE,RELA,RELB
IL-8 Signaling	5.13	−2.82	DIRAS3,HBEGF,IKBKE,IRAK2,KDR,LIMK2,MAP2K1,MMP9,NFKBIA,NFKBIE,PIK3C2B,PIK3R6,PRKCD,PTGS2,RALA,RAP2A,RASD1,RELA,TEK,VEGFA,VEGFC
